# Characterization and potential lipid-lowering effects of lactic acid bacteria isolated from cats

**DOI:** 10.3389/fmicb.2024.1392864

**Published:** 2024-04-24

**Authors:** Shukun Liang, Yanhua Kang, Ya Zhao, Jintao Sun, Xiumin Wang, Hui Tao, Zhenlong Wang, Jinquan Wang, Yougang Zhong, Bing Han

**Affiliations:** ^1^Key Laboratory of Feed Biotechnology of Ministry of Agriculture and Rural Affairs, Institute of Feed Research, Chinese Academy of Agricultural Sciences, Beijing, China; ^2^School of Veterinary Medicine, China Agricultural University, Beijing, China

**Keywords:** lactic acid bacteria, cholesterol, probiotics, microbiota, high-fat diet

## Abstract

**Introduction:**

This study aimed to study the characterization and the potential lipid-lowering effects of new isolated lactic acid bacteria from the feces of healthy adult cats.

**Methods:**

We collected 85 cat fecal samples, isolated, screening lactic acid bacteria strains from samples, and investigated their *in vitro* and *in vivo* biological properties.

**Results:**

A total of 221 lactic acid bacteria strains were isolated from 85 cat fecal samples. Sixteen strains with calcium dissolution rings greater than 1 mm were identified and selected for further characterization. Three lactic acid bacteria strains, *Lactobacillus plantarum* L-27-2, *Pediococcus lactis* L-14-1, and *Enterococcus faecium*, were identified as showing the most promising rates of cholesterol degradation (greater than 20%) and bacteriostatic radius (over 15 mm). These three strains exhibited robust growth and adherence to epithelial cells, along with adaptability to low pH (greater than 70%) and high bile salt conditions (greater than 60%), and remarkable cholesterol degradation and anti-pathogen activity. Sixteen mice were fed a high-fat diet (HFD) from 4 to 8 weeks of age, while a control group of the same size received a normal diet (ND). At 8 weeks of age, serum, feces and adipose tissue were collected. The results showed that, compared with mice fed an HFD diet alone, all mice fed an HFD diet plus lactic acid bacteria could decrease weight gain. *P* < 0.05 and the pathological changes of adipose tissue were alleviated. In addition, mice fed L-14-1 and F203 showed abdominal fat accumulation decreased (*P* < 0.05). Mice fed L-27-2 showed serum and liver triglyceride (TG) decreased (*P* < 0.05) and mice fed F203 showed serum high density lipoprotein cholesterol (HDL-C) increased (*P* < 0.01). mice fed L-27-2 and L-14-1 showed inflammatory cytokines (IL-6) was decreased (*P* < 0.01) Analysis of the fecal microbiota of mice fed these three lactic acid bacteria strains revealed alterations in the gut microbial community. There were common changes in intestinal microbes in mice fed these three lactic acid bacteria: (1) *Bacteroides* decreased; (2) *Myxococcus* increased; (3) *Lachnoclostridium* decreased. The microbes mentioned are all part of the core intestinal flora.

**Discussion:**

This study provided three potential lactic acid bacteria for alleviating animal obesity and inflammation.

## 1 Introduction

At present, a major concern in animal clinics is the increase in the incidence of nutritional metabolic diseases, including obesity, diabetes, pancreatitis, and others, which have serious health implications for domestic animals. The prevalence of obese animals in the general population has been steadily increasing (German, 2006). The primary method used to manage animal weight is by limiting energy intake. However, due to the lack of effective preparations to reduce cholesterol absorption, the occurrence of obesity in animals remains difficult to control (German, 2006; Linder and Mueller, 2014). In addition, these nutritional metabolic diseases can also lead to reduced resistance to pathogenic bacteria in the intestine. Pathogens such as *Salmonella*, *Escherichia coli*, and *Staphylococcus aureus* can trigger varying degrees of inflammation and cause diarrhea in the body, exacerbating conditions in obese animals (Takemori et al., 2020). Therefore, there is a pressing need to discover drugs or preparations that can alleviate animal obesity over an extended period of time.

Probiotic preparations are commonly consumed in daily diets, with the most common probiotic preparations consisting primarily of lactic acid bacteria (Mann et al., 2021). The lactic acid bacteria have been found to be essential in maintaining the balance of gut microbial ecosystems. The primary role of lactic acid bacteria in animals is to regulate the composition and metabolism of intestinal microbes (Fernández et al., 2018). Current clinical applications of lactic acid bacteria include reducing intestinal cholesterol absorption, reducing lipid deposition in the body, and inhibiting the growth of pathogenic bacteria (Lin et al., 2020; Yao et al., 2022).

There is a paucity of excellent strains of lactic acid bacteria that can reduce cholesterol absorption and inhibit the growth of pathogenic bacteria, while also showing excellent growth characteristics such as rapid propagation, easy culture, and convenient storage. The purpose of this study was to screen lactic acid bacteria strains with lipid-lowering effect from cat feces, evaluate the probiotic characteristics and cholesterol-lowering potential of isolated strains through *in vitro* experiments, and evaluate the *in vivo* characteristics of lactic acid bacteria by feeding mice with a high-fat diet (HFD), including the potential to reduce obesity and inflammation caused by HFD.

## 2 Materials and methods

### 2.1 Sample collection and screen

Fecal samples were collected from a group of 85 healthy cats aged 2–4 years, fully immuned to deworming, disease-free and in good health. The weight of the cats ranged from 2.5 to 4 kg. Samples were collected after the cat had defecated, then transferred to a 50 ml sterile centrifuge tube and subjected to a rapid freezing protocol using liquid nitrogen, subsequently stored at −80°C. After thawing the frozen fecal samples at room temperature for 15 min, weigh 1 g of the samples and put them in 9 ml of MRS liquid culture medium (1% peptone, 0.5% beef powder, 2% glucose, 0.4% yeast barm) (Solarbio, Beijing, China) at 37°C for 24 h. Then, pick out the liquid culture medium with sterile inoculation ring and coated it on MRS solid culture medium (1% agar, w/v) containing 2% CaCO_3_, and let it stand at 37°C for 24 h. After that, the single colony with obvious dissolution circle greater than 1 mm was selected to be enriched and cultured in MRS liquid medium, and then stood at 37°C for 24 h. All growth environments were micro-aerobically and we provided micro-aerobic environments through standing cultures. After that, single colonies were selected again and cultured in MRS liquid. The purification was repeated twice. After that, 80% glycerol was used to preserve bacteria and frozen at −80°C.

### 2.2 Determination of cholesterol degradation ability

Bacteria was inoculated at 1% inoculum into MRS liquid culture medium at 37°C for 24 h, 1% inoculum from which was inoculated into MRS liquid culture medium with cholesterol (0.1 g/L), and after 24 h, 0.2 ml of bacteria liquid was taken into 10 ml centrifuge tube. A total of 4.8 ml absolute ethanol was then added into the centrifuge tube, still for 15 min, and then centrifuged at 6,659 × *g* for 10 min. Aspirate 2 ml of supernatant into a test tube, then 2 ml of ferric alum chromogenic agent solution was added. After cooling to room temperature, the OD of liquid was measured at 560 nm with an enzyme-labeled instrument within 15–90 min. Using sterile cholesterol standard solutions with cholesterol concentrations of 0.02, 0.04, 0.06, 0.08, and 0.10 mg/ml, respectively, the standard curve was drawn with OD 560 nm value and standard concentration. The cholesterol concentration in the experimental group was recorded as Cb, and the cholesterol concentration in the control group was recorded as Ca.

Cholesteroldegradationrate/%=(Ca-Cb)/Ca×100

### 2.3 Determination of bacteriostasis

The lactic acid bacteria strains in the logarithmic growth period were diluted with physiological saline to a bacterial solution with a concentration of 10^8^ CFU/ml, and the bacteriostatic effect of the lactic acid bacteria strains on pathogenic bacteria was detected by agar diffusion oxford cup. *E. coli ATCC 14028, S. aureus ATCC 43300, Salmonella CVCC 3377* were selected as pathogenic bacteria. All pathogens were grown in NA medium (1% peptone, 0.3% beef powder, 0.5% NaCl) (Solarbio, Beijing, China) and incubated at 37°C for 24 h. All pathogens were grown in NA medium (1% agar, w/v). Then, the bacterial solution of each lactic acid bacteria strains (10^8^ CFU/ml) were used to fill a 6 mm Oxford cup previously punched on LB agar plates. All plates were incubated at 37°C for 24 h, and the radius of the antibacterial circle around the wells were measured. According to the size of the inhibition zone, it can be divided into three types: no inhibition (*r* = 0 mm), inhibition (*r* = 0–10 mm), and obvious inhibition (*r* > 10 mm), and lactic acid bacteria with inhibition zone radius >15 mm were screened, and sterile MRS liquid culture medium was used as blank control.

### 2.4 16S rRNA sequencing and phylogenetic tree construction

The genomic DNA of lactic acid bacteria was extracted by bacterial genome extraction kit (Solarbio, Beijing, China), and the target fragment was amplified by 16S rDNA universal primers 27F and 1492R. The purified and recovered PCR products were sent to Shenggong Bioengineering Co., Ltd. for sequencing. The sequencing results were compared with BLAST homology on NCBI website. The nucleic acid sequence of the lactic acid bacteria model strain with the highest homology was selected for homology analysis, and constructed phylogenetic tree by MEGA software. Combined with biological characteristics and alignment of the 16S rRNA gene sequence (SEQ ID NO: 1), the strain was determined.

### 2.5 Identification of physiological and biochemical properties

Single colony of each isolate was selected and mixed it evenly in sterile PBS solution to make a solution with Maxwell’s ratio turbidity of 2. A total of 400 μl of liquid was taken into API liquid culture medium (Meria French), mixed evenly, and then 200 μl of liquid was inoculated onto glucose fermentation test strips, such as glycerol and erythritol, and incubate at 37°C for culture, observed and recorded changes in test strips.

### 2.6 Growth curve determination

Activated bacterial liquid was inoculated into MRS liquid culture medium with 1% inoculation amount, and cultured at 37°C for 48 h. The OD 600 nm value and pH value of bacterial liquid were determined every 4 h, and the growth curve and acid production curve of the strain were drawn.

### 2.7 Acid resistance and bile salt tolerance

MRS liquid medium was adjusted to pH 2, 3, and 4 by HCL (98%), respectively, and each strain was inoculated in the medium according to the inoculation amount of 0.01%. After 2 and 4 h of culture, 100 μl above bacterial liquid was inoculated at solid medium and cultured at 37°C for 24 h, and then the number of live bacteria was counted to determine the acid resistance of each strains (Yang E. et al., 2018). Similarly, the above culture medium were inoculated with bile salt content of 0%, 0.15%, 0.3%, and 0.6%, and the number of living bacteria was counted after 24 h at 37°C to determine the bile salt tolerance of lactic acid bacteria, and the strains with tolerance to bile salt concentration greater than 0.3% were screened out.

### 2.8 Antibiotic sensitivity

Using disk diffusion test (K-B method), depending on the size of the bacteriostatic ring, it was determined whether different isolates were sensitive to common antibacterial drugs such as amoxicillin clavulanate potassium, ceftiofur, cefquinome, and doxycycline. Under aseptic conditions, single colonies were picked from sterilized inoculation rings and inoculated in MRS medium for 24 h at 37°C. Each plate was coated with 100 μl of bacterial liquid, which was dripped on the surface of MRS solid culture medium. After uniform coating, the drug-containing paper was removed by sterile tweezers and stuck to the surface of the culture medium. After standing for a period of time, it was cultured in a constant temperature box at 37°C for 24 h (Kang et al., 2020). The bacteriostatic results were observed and the radius of bacteriostatic circle of each tablet group was measured. At the same time, according to the Experimental Specification for Antimicrobial Drug Sensitivity, the drug sensitivity effect was judged, and the results were divided into three types: sensitive (*r* > 15 mm), intermediate (*r* = 15–10 mm), and drug resistance (*r* < 10 mm), and antibiotic sensitivity characteristics of various lactic acid bacteria were detected.

### 2.9 Cell adhesion capacity

The adhesion potential of lactic acid bacteria was investigated using epithelial cell lines. Cells were grown in an environment supplemented with 10% fetal bovine serum at 37°C and 5% carbon dioxide, and monolayer cells were prepared in a six-hole tissue culture plate. Epithelial cell lines were inoculated at a concentration of 5 × 10^8^ and incubated in a 5% carbon dioxide incubator at 37°C. Cells grown in an environment of 5.0 × 10^8^ lactic acid bacteria were added and washed twice with PBS. The number of lactic acid bacteria adhered to 20 cells in the field of random oil mirror was observed by microscope, and the adhesion capacity of lactic acid bacteria was calculated by number, which was divided into non-adhesion (less than one bacteria adhered to 20 cells), adhesion (1–8 bacteria adhered to 20 cells) and strong adhesion (more than 8 bacteria adhered to 20 cells) (Shukla and Goyal, 2014).

### 2.10 Animals and experimental design

All animal experiments were approved by the Laboratory Animal Ethical Committee and its Inspection of the Institute of Feed Research of CAAS (IFR-CAAS-20230301). Twenty healthy 4 week old male BAL b/c mice raised at 22 ± 1°C and 55 ± 10% humidity on a 12 h light/dark cycle on the pilot base of the Chinese Academy of Agricultural Sciences. After 1 week of adaptation, they were randomly divided into five groups, with four mice in each group. They were L-27-2 group (supplemented with *Lactobacillus plantarum* L-27-2 in HFD), L-14-1 group (supplemented with *Pediococcus* sp. L-14-1 in HFD), F203 group (supplemented with *Enterococcus faecium* F203 in HFD), M group (HFD model group), and CON group (normal diet group). The experiment was conducted for 28 days, with four mice per cage. All mice were fed with an unrestricted amount of food and water daily, and no additional antibiotics or probiotics were given during the experiment. Group L-27-2, group L-14-1, group F203, and group M were fed a HFD (60% fat, 20% protein, and 20% carbohydrate), while group CON was fed a normal diet (ND) (5% fat, 18% protein, and 77% carbohydrate). Mice were given the corresponding lactic acid bacteria freeze-dried powder at a dose of 10^9^ CFU/kg daily, diluted with sterile PBS, gastric administration every morning and M group and CON group were given the same amount of sterile PBS for 28 days. Total intake and weight were recorded every 14 days. The mice were euthanized by neck removal. Blood samples were collected from the eyeball and stored at −20°C. Colonic contents were rapidly frozen in liquid nitrogen, and then stored to −80°C. In addition, fecal samples and other issues were stored at −20°C. Fat samples were soaked in tissue fixative and stored at 4°C to observe the effects of lactic acid bacteria on body weight, abdominal fat, liver, serum immunoglobulin, lipid metabolism-related proteins, and fecal microorganisms in mice induced by the HFD.

### 2.11 Body weight and related organ indexes

Feed intake and weight of each mice were measured and recorded on day 0, 14, and 28 after the start of the experiment. On the 28th day, the mice were dissected, liver and abdominal fat washed with sterile saline, and filter paper was used to absorb water, record appearance, weigh, and calculate liver and obesity indices.

L⁢i⁢v⁢e⁢i⁢n⁢d⁢e⁢x=(o⁢r⁢g⁢a⁢n⁢w⁢e⁢i⁢g⁢h⁢t/b⁢o⁢d⁢y⁢w⁢e⁢i⁢g⁢h⁢t)×100

O⁢b⁢e⁢s⁢i⁢t⁢y⁢i⁢n⁢d⁢e⁢x=(a⁢b⁢d⁢o⁢m⁢i⁢n⁢a⁢l⁢f⁢a⁢t⁢w⁢e⁢i⁢g⁢h⁢t/b⁢o⁢d⁢y⁢w⁢e⁢i⁢g⁢h⁢t)×100

### 2.12 Determination of protein related to lipid metabolism and inflammatory factor in serum

Mice orbital blood was collected in a centrifuge tube, and the whole blood was kept at room temperature for 30 min, so as not to shake violently to avoid hemolysis. After the whole blood was naturally solidified, serum was collected. Inflammatory cytokines 6 (IL-6) and indexes related to lipid metabolism [total cholesterol (TC), triglyceride (TG), high-density lipoprotein cholesterol (HDL-C), low density lipoprotein (LDL-C)] were detected by ELISA. The serum was put on ice for later use. Competitive ELISA kit (Jiangsu Yancheng Jiangsu Meimian Industrial Co., Ltd., Yancheng, China) was used for detection. First, the standard was diluted to draw the standard curve. The test sample and standard competitive antigen (HRP-labeled) were added, gently shaken and stirred, and stood at room temperature in the dark for 60 min. Then the plate was washed five times and pat dry. TMB was added to stop color development, and the absorbance value (OD 450 nm) of each well was measured. If the detection result was valid within 15 min, draw a standard curve with OD 450 nm value and standard concentration. Then the concentrations of IL-6, TC, TG, HDL-C, and LDL-C in the sample were determined by comparing the external diameter of the sample with the standard curve. Because of the use of competitive ELISA detection kits, the OD 450 nm value was negatively correlated with sample concentration.

### 2.13 Pathological and histological analysis of fat

Fat samples were fixed in tissue fixative, sliced into 4 μm and baked at 65 rve. Xylene solution A and xylene solution B were added sequentially for 1 min, slicing for dewaxing, 100% ethanol was added sequentially for 10 min, 95% ethanol for 1 min, 80% ethanol for 1 min and 70% ethanol for 1 min, and xylene was removed by eluting with water. Then the hematoxylin dye solution was added for 3 min and rinse with water. One percent hydrochloric acid alcohol was added for 30 s and washed with water. One percent ammonia water was added for 30 s and rinsed with water. Eosin dye solution was added for 2 min and rinsed with water. A total of 70% ethanol, 80% ethanol, 95% ethanol, and 100% ethanol were added each for 1 min in turn. Blow dry, pass through xylene solution and blow dry again. The film was read in the mirror.

### 2.14 Detection of cholesterol and triglycerides in the liver

Liver tissue (0.1 g) of mice were collected, extracted in RIPA lysis buffer containing protease/phosphatase inhibitor by tissue homogenizer, and homogenized at 4,722 × *g* for 30 s, 4 times for 2 min. The homogenate samples were centrifuged at 10,625 × *g* at 4°C for 3 min, then the supernatant was separated and stored at 4°C. Lipid metabolism related indexes (TC and TG) were detected by ELISA. The ELISA kit assay (Jiangsu Meimian Industrial Co., Ltd., Yancheng, Jiangsu) was used to test. First, the standard was diluted and used to draw the standard curve. The test sample and standard competitive antigen (HRP-labeled) were added, gently shaken and stirred, and stood at room temperature in the dark for 60 min. Then the plate was washed five times and pat dry. TMB was added to stop color development, and the absorbance value (OD 450 nm) of each well was measured. If the detection result was valid within 15 min, draw a standard curve with OD 450 nm value and standard concentration. Then the concentrations of TC, TG in the sample were determined by comparing the outer diameter of the sample with the standard curve.

### 2.15 Extraction of fecal DNA

Microbial genomic DNA was extracted from 20 samples of colon contents by a E.Z.N.A. Mag-Bind Soil DNA Kit (Omega, M5635-02, USA) and Quibit dsDNA HS (Thermo, USA) was used to test the concentration of DNA samples. The extracted DNA samples were stored in a freezer at −80°C and used for the PCR amplication.

### 2.16 PCR amplication

The sequence of forward primer was CCTACGGGNGGCWGCAG, and the reverse primer was GACTACHVGGGTATCTAATCC. The PCR reaction conditions were as follows: 94 ermo, USA, 5 cycles at 94°C for 30 s, 45°C for 20 s, 65°C for 30 s; 20 cycles at 94°C for 20 s, 55°C for 20 s, 72°C for 30 s; 72°C for 5 min. PCR products were purified and quantified bt QIAquick gel extraction kit (QIAGEN, Hilden, Germany) and Quant-iT PicoGreen dsDNA assay kit (Life Technologies, Carlsbad, CA, USA), respectively. The extracted DNA samples were sequenced for 16S rDNA in Sangon Biotech (Shanghai) Co., Ltd.

### 2.17 Data analysis

Gut microbiota evaluation was made using QIIME and the R package 3.5.1, whereas Alpha diversity indices and bacterial abundance data of different groups were compared by performing the Kruskal–Wallis test followed by pairwise Mann–Whitney U comparison. Then, resulting *P*-values were corrected with the Bonferroni method. The alpha-diversity analysis was conducted to investigate the microbial diversity using Shannon diagram, and beta-diversity analysis was conducted to investigate the structural variation of microbial communities across samples using UniFrac distance metrics and visualized via principal coordinate analysis (PCoA). Besides, difference comparison was used to identify features with significantly different abundances between groups using STAMP (version 2.1.3) and LefSe (version 1.1.0). Correlation coefficients and *P*-values between communities/OTUs were calculated using SparCC (version 1.1.0). Except the 16S rRNA data has been described in detail, the data obtained in this study are presented as the mean ± SEM. Besides, all statistical analyses were carried out by applying the IBM SPSS Statistics software 19.0 for Windows, while differences between two groups were assessed using the unpaired two-tailed Student’s *t*-test. In addition, more than two groups were evaluated by a one-way analysis of variance (ANOVA) followed by Newman–Keuls *post-hoc* tests. Here, it should be noted that a *P*-values of *P* < 0.05 was considered statistically significant. The following *P*-values were used: *P < 0.05; **P < 0.01; ***P < 0.001; ****P < 0.0001.

## 3 Results

### 3.1 Screening of strains with cholesterol degrading ability and bacteriostatic ability in feces

**FIGURE 1:**
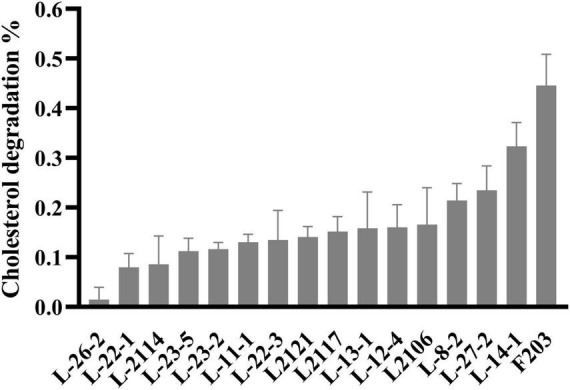
Cholesterol degradation curve. Cholesterol degradation %: (Ca – Cb) / Ca × 100, Ca = 0.0815 mg/ml.

A total of 221 lactic acid bacteria strains were isolated from 85 cats for calcium dissolving rings, and 16 strains with calcium dissolving rings larger than 1 mm were selected (Supplementary Table 1). The colony morphology was observed by Gram staining and microscopic examination (Supplementary Table 2). After that, the ability of 16 strains to degrade cholesterol was measured, and 8 strains with more than 15% of the ability to degrade cholesterol were screened (Figure 1). The strains that inhibited *Salmonella, S. aureus*, and *E. coli* were screened, and nine strains with antibacterial circle radius greater than 15 mm were screened (Table 1). A total of six strains with both cholesterol-degrading and antibacterial abilities were selected, and three strains (L-27-2, L-14-1, and F203) with better ranking were selected to test other growth performance.

**TABLE 1:** The ability of inhibit pathogenic bacteria.

Strain number	Antibacterial Circle (mm)^a^		
	***S. aureus ATCC 43300***	***Salm CVCC 3377***	***E. coli ATCC 14028***
L-11-1	13.03 ± 0.15	14.80 ± 0.15	14.93 ± 0.15
L-12-4	13.16 ± 0.25	16.20 ± 0.35	14.20±0.52
L2121	13.90 ± 0.28	14.83 ± 0.41	14.93 ± 0.41
L-22-3	14.06 ± 0.15	15.83 ± 0.15	14.90 ± 0.41
L-8-2	15.06 ± 0.25	16.06 ± 0.25	14.06 ± 0.32
L-23-2	14.83 ± 0.40	15.86 ± 0.65	14.73 ± 0.15
L-23-5	14.93 ± 0.25	15.93 ± 0.25	14.63 ± 0.20
L2117	15.50 ± 0.20	15.56 ± 0.25	15.96 ± 0.15
L-26-2	15.26 ± 0.36	17.06 ± 0.28	15.06 ± 0.32
L-14-1	15.00 ± 1.00	16.67 ± 0.58	16.33 ± 1.15
L-13-1	14.06 ± 0.15	18.10 ± 0.20	15.96 ± 0.15
L2106	18.30 ± 0.30	15.93 ± 0.10	14.10 ± 0.20
L-22-1	15.86 ± 0.26	18.73 ± 0.15	14.70 ± 0.15
F203	15.43 ± 0.15	18.46 ± 0.15	16.86 ± 0.32
L-27-2	16.90 ± 0.36	17.60 ± 0.26	17.23 ± 0.35
L2114	15.93 ± 0.41	18.63 ± 0.35	18.63 ± 0.20

^a^r = Mean ± SEM.

### 3.2 16S rRNA sequencing and phylogenetic tree construction

**FIGURE 2:**
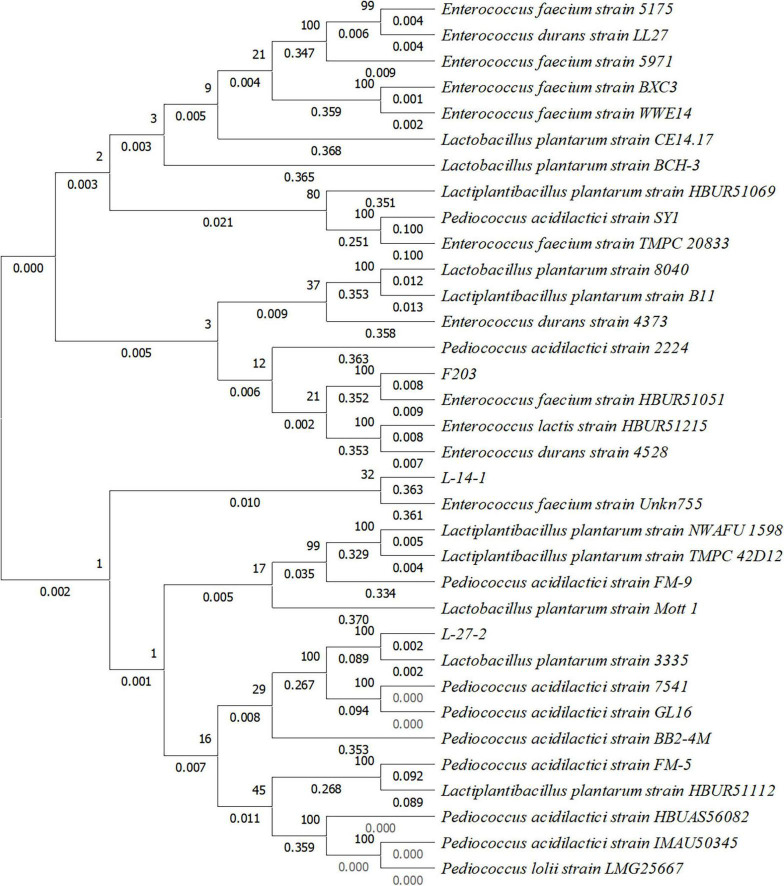
Phylogenetic tree constructed based on 16S rRNA gene sequence of three lactic acid bacteria strains isolated from cat feces.

Neighbor-Joining method was used to draw phylogenetic trees (Figure 2). Evolutionary distance was calculated by the *p*-distance method, and the unit was the number of base differences at each locus. This analysis involved 34 nucleotide sequences including a noncoding sequence with codon at base 1, base 2, and base 3. Removed all ambiguous positions from each sequence pair, and there were 998 locations in the final dataset. According to these 998 loci, the difference in the base number of each strain was shown at the nodes of the tree. Based on the baseline sequence and phylogenetic tree results, the fecal strains L-27-2, L-14-1, and F203 were identified as *L. plantarum, Pediococcus* sp., and *E. faecium*. The login number in Genbank are: OR919791, OR919790, and OR919789.

### 3.3 Carbohydrate fermentation ability

The physiological and biochemical characteristics of three isolated strains were tested. It was found that L-27-2, L-14-1, and F203 had the ability to ferment common monosaccharides and polysaccharides, such as glucose and sucrose, of which F203 could ferment most types of carbohydrates (23 types), and at the same time, the color reaction results of all carbohydrates were integrated. The strains L-27-2, L-14-1, and F203 were identified as *L. plantarum, Pediococcus* sp., and *E. faecium*, respectively. The fermentation properties of L-27-2 were different from the bacteria found by others, which could ferment arabinose but had no obvious effect on ribose (Cui et al., 2021). Compared to *Lactococcus lactis* strains detected by Passerini et al. (2013), the fermentation properties of L-14-1 showed an additional ability to utilize lactose and xylose. There are few studies on carbohydrate fermentation by *E. faecium* (Halstead et al., 2015; Kim et al., 2020). Comparing F203 with Kim MA, we found that *E. faecium* has the ability to ferment sucrose, and it also has the ability to ferment lactose and maltose (Table 2).

**TABLE 2:** Physiological and biochemical characteristics.

Physiological and biochemical experiments	Trial result (±)*			Physiological and biochemical experiments	Trial result (±)		
	**L-27-2**	**L-14-1**	**F203**		**L-27-2**	**L-14-1**	**F203**
Glycerol	−	−	−	Salicin	+	+	+
Erythritol	−	−	−	D-cellobiose	+	+	+
D-arabinose	−	−	−	D-maltose	+	−	+
L-arabinose	+	−	+	D-lactose	−	+	+
D-ribose	+	+	+	D-melibiose	+	+	+
D-xylose	−	+	+	Sucrose	+	+	+
L-xylose	−	+	−	D-trehalose	+	−	+
Ribitol	−	−	−	Inulin	−	−	−
Methyl-losee (±)d biochemi	−	−	−	D-melezitose	+	−	+
D-galactose	+	−	+	D-raffinose	−	+	−
D-glucose	+	+	+	Starch	−	−	−
D-fructose	+	+	+	Glycogen	−	−	−
D-mannose	+	+	+	Xylitol	−	−	−
L-sorbose	−	+	−	D-gentianodisacCharide	+	+	+
L-rhamnose	−	−	−	D-toulon sugar	+	−	+
Euonymol	−	−	−	D-Lysol	−	−	−
Inositol	−	−	−	Tagatose	−	+	+
Mannitol	+	−	+	D-fucose	−	−	−
Sorbitol	−	−	−	L-fucose	−	−	−
Methyl-e sugarcCharide	+	−	−	D-arabic alcohol	−	−	−
Methyl-alcoholharidehemica	−	−	−	L-arabic alcohol	−	−	−
N-acetylglucosamine	+	+	+	Potassium gluconate	−	−	−
Amygdalin	+	+	+	Potassium 2-ketogluconate	−	−	−
Arbutin	+	+	+	Potassium 5-ketogluconate	−	−	−
Seven Ling Ye ferric citrate	+	+	+				

*+: API liquid medium changed from purple to yellow-green; −: the color of API liquid medium is still purple and will not change.

### 3.4 Growth curve determination

**FIGURE 3:**
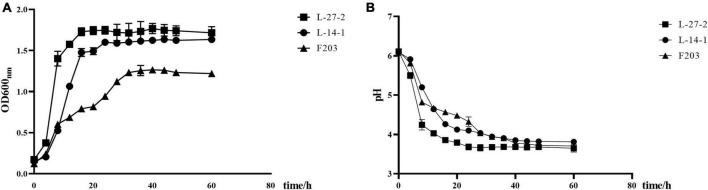
The growth characteristics. **(A)** Growth curve of three lactic acid bacteria strains. **(B)** Acid production curve of three lactic acid bacteria strains.

The growth performance of L-27-2, L-14-1, and F203 was investigated. It was found that L-27-2, L-14-1, and F203 all reached a stable stage after 18 h of growth, among which L-27-2 and L-14-1 could reach higher biomass, with an OD 600 nm of about 1.8, while F203 had less biomass and lower OD 600 nm (Figure 3A). L-27-2, L-14-1, and F203 can all produce acid with a pH value of about 3.5(Figure 3B), and this value remains unchanged after the strains entered the growth stable period, showing a lower pH compared to the former research (pH = 4.1), which may be related to the higher ability of these three strains to produce SCFA (LeBlanc et al., 2004; Sun et al., 2023), and the time to enter the growth stable period was obviously earlier than other lactic acid bacteria. These growth characteristics can make L-27-2, L-14-1, and F203 better stored and clinically applied (Wang et al., 2022).

### 3.5 Acid and bile salt tolerance of strain

Due to the acidic environment and high bile salt environment in the gastrointestinal tract, a good probiotic preparation needs to have good acid and bile salt resistance (Holzer, 2007; Mulaw et al., 2019; Naqqash et al., 2022). The acid resistance and bile salt resistance of L-27-2, L-14-1, and F203 were determined. It was found that L-27-2, L-14-1, and F203 maintained high vitality after 4 h in pH 2 environment, and survival rates reached 93.71%, 96.80%, and 70.17%. L-27-2 was well adapted to pH 3, and the survival rate of L-14-1 and F203 at pH 3 was 96.96% and 86.21%, respectively. The pH 4 environment was conducive to the growth of L-27-2 and L-14-1, and the survival rate of F203 was 91.56% at pH 4 (Table 3), which showed the same trend as Reuben et al.’s (2020) experimental results. In addition, survival rates for L-27-2, L-14-1, and F203 were 69.76%, 68.27%, and 60.23% at 0.6% bile salt concentration, respectively, and survival rates increased with the decrease of bile salt concentration. The results showed that L-27-2, L-14-1, and F203 could tolerate acid environment and high bile salt (0.6%) environment well (Table 4). Because the gastrointestinal pH was more than 2 and the salt concentration in the intestinal lining was less than 0.3%, it could tolerate the acidic environment with pH 2 and the bile salt environment of 0.6%, which proved that L-27-2, L-14-1, and F203 could exist well in the intestinal tract and would not lead to the reduction of biomass due to the special environment in the intestinal tract (Mukherjee et al., 2013; Winston and Theriot, 2020).

**TABLE 3:** Survival rate of three lactic acid bacteria strains in different pH environments (%).

Strain number	pH^a^			
	**2**	**3**	**4**	**7 (Control)**
L-27-2	93.71%	100%	100%	100%
L-14-1	96.80%	96.96%	100%	100%
F203	70.17%	86.21%	91.56%	100%

^a^Survival rate = Mean (pH = 2, 3, or 4) / Mean (pH = 7).

**TABLE 4:** Survival rate of lactic acid bacteria at different bile salt concentrations (%).

Strain number	Bile salt concentration (%)^a^			
	**0 (Control)**	**0.15**	**0.3**	**0.6**
L-27-2	100%	89.66%	90.53%	69.76%
L-14-1	100%	96.96%	80.82%	68.27%
F203	100%	80.60%	68.24%	60.23%

^a^Survival rate = Mean (pH = 2, 3, or 4) / Mean (pH = 7).

### 3.6 Antibiotic sensitivity of lactic acid bacteria strains

The antibiotic sensitivity of three strains was tested, and it was found that all of these strains showed good sensitivity to common clinical antibiotics (Table 5), indicating that these three strains could not easily cause drug resistance (Levy and Marshall, 2004; Nowakiewicz et al., 2021).

**TABLE 5:** Survival rate of lactic acid bacteria at different bile salt concentrations (%).

Antibiotic	Radius of inhibition zone (average ± standard deviation mm)^a^		
	**L-27-2**	**L-14-1**	**F203**
erythromycin	16.16 ± 0.35	17.23 ± 0.25	16.07 ± 0.15
Minocycline	16.06 ± 0.23	15.10 ± 0.10	22.07 ± 0.15
ceftriaxone	15.23 ± 0.15	15.47 ± 0.42	16.47 ± 0.47
Pearl necklace	16.33 ± 0.15	16.23 ± 0.15	17.97 ± 0.32
Cefozolin	20.06 ± 0.25	18.13 ± 0.58	19.93 ± 0.37
Piperacillin	20.06 ± 0.15	20.40 ± 0.26	24.87 ± 0.35
Carbenicillin	20.10 ± 0.26	22.13 ± 0.06	23.30 ± 0.26
Ampicillin	16.20 ± 0.30	18.23 ± 0.15	22.97 ± 0.32
Penicillin	21.13 ± 0.21	21.13 ± 0.06	24.93 ± 0.15

^a^ r = Mean ± SEM.

### 3.7 Adhesion capacity of lactic acid bacteria strains

**FIGURE 4:**
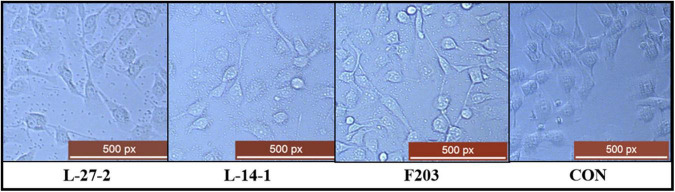
Adhesion of three strains to epithelial cells. L-27-2, L-14-1, and F203 adhere to cells, while CON is a cell without bacterial adhesion.

The adhesion capacity of L-27-2, L-14-1, and F203 to epithelial cell lines was shown in the Figure 4. At least 20 L-27-2 cells adhere to a single epithelial cell, and at least 10 L-14-1 and F203 cells adhere to a single epithelial cell (Figure 4). These three strains of lactic acid bacteria showed good adhesion to epithelial cells, indicating that lactic acid bacteria can adhere to intestinal epithelial cells well, which also made them more capable of competing with pathogenic bacteria (Uraipan and Hongpattarakere, 2015; Sirichokchatchawan et al., 2018; Yang J. et al., 2018), becoming the dominant bacteria in the intestinal tract and improving the intestinal environment (Salonen et al., 2009; Liu et al., 2018, 2021).

### 3.8 Lactic acid bacteria decreased weight gain induced by the HFD in mice

**FIGURE 5:**
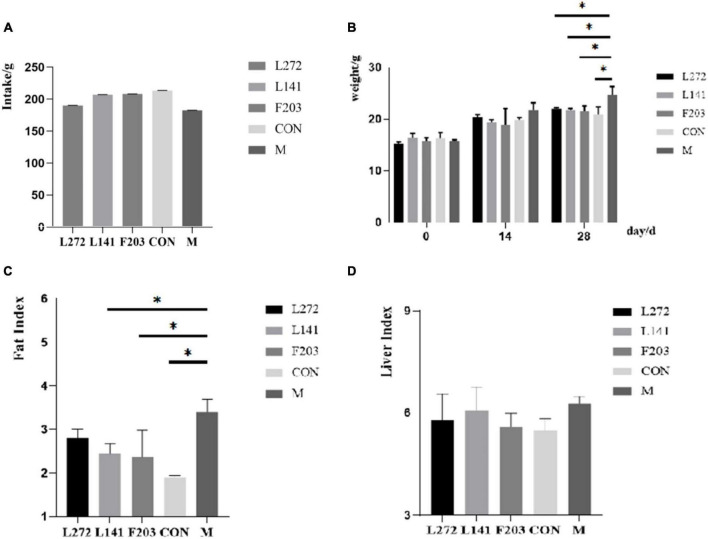
Weight-related indexes in HFD mice. **(A)** Total intake of mice. **(B)** Weight of mice at 0, 14 and 28 days. **(C)** Fat index of mice. **(D)** Organ index of mice. *p < 0.05.

Studies have shown that the HFD could lead to obesity in mice (Kim et al., 2023). By feeding mice with the HFD, it was observed that the intake did not decrease significantly for 28 days after feeding with HFD. The results showed that the weight and obesity index increased significantly (*P* < 0.05) and the liver index increased (*P* > 0.05). Supplementation with L-27-2, L-14-1, and F203 could slow weight gain, reduce fat accumulation, and reduce liver index (*P* > 0.05) and fat index (*P* < 0.05) in mice fed with HFD (Figures 5A–D). However, the effect of L-27-2 in lowering the obesity index and the tendency of the three strains to lower liver weight was not significant, which may be related to the short duration of the trial. According to former research, lactic acid bacteria have obvious effects on reducing obesity and weight (Crovesy et al., 2017; Khare and Gaur, 2020; Zhong et al., 2022). This conclusion was consistent with our results which confirm the potential of L-27-2, L-14-1, and F203 to reduce body weight gain in animals.

### 3.9 Lactic acid bacteria modulated lipid levels in mice fed the HFD

**FIGURE 6:**
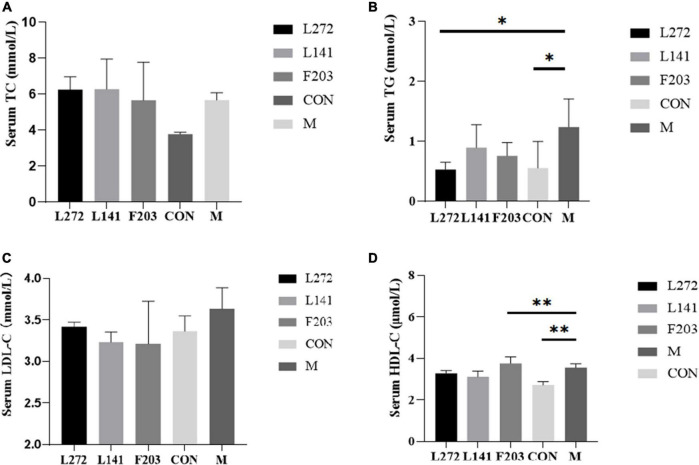
Blood lipid-related indexes in mice. **(A)** Serum TC content of mice on 28th day. **(B)** Serum TG content of mice on 28th day. **(C)** Serum LDL-C content of mice on 28th day. **(D)** Serum HDL-C content of mice on 28th day. *p < 0.05, **p < 0.01.

To further confirm the effects of L-27-2, L-14-1, and F203 on blood lipids, the blood lipid content (TG, TC, LDL-C, and HDL-C) of mice was biochemically analyzed. The results showed that the increase in TC, TG, and LDL-C was closely related to hyperlipidemia, obesity, and other diseases (Retterstøl et al., 2018; Webb et al., 2022; Duan and Li, 2023). However, all three strains can reduce TG in serum, however, TC and LDL-C showed only a decreasing tendency, and their differences with M group were not significant (*P* > 0.05) (Figures 6A, C). The serum TG content of L-27-2 decreased significantly (*P* < 0.05), while that of L-14-1 and F203 decreased (*P* > 0.05) (Figure 6B). In addition, feeding F203 could also increase the concentration of HDL-C in the blood (*P* < 0.01) (Figure 6D), and the increase of HDL-C could promote the reverse transport of cholesterol and reduce lipid accumulation. These results indicated that lactic acid bacteria L-27-2, L-14-1, and F203 could reduce the increase in blood lipid in mice induced by the HFD, thereby reducing the occurrence of nutrition-related metabolic diseases such as hyperlipidemia and diabetes.

### 3.10 Lactic acid bacteria relieved inflammation in mice fed the HFD

**FIGURE 7:**
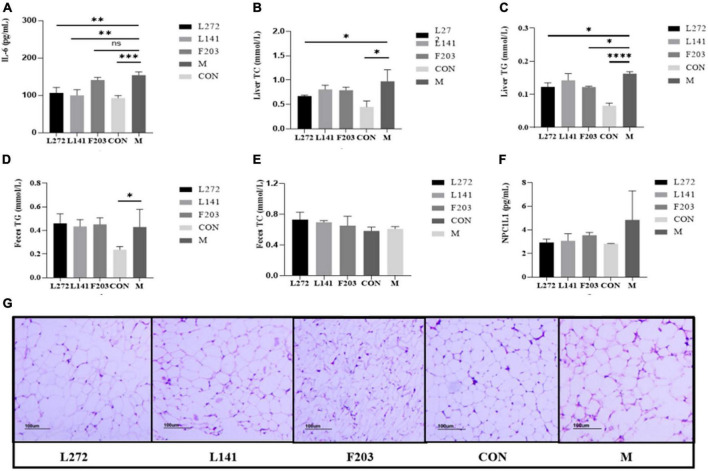
Lipid and inflammation related indexes in mice. **(A)** Blood proinflammatory factor (IL-6) content in mice. **(B)** TC content in the liver of mice. **(C)** TG content in the liver of mice. **(D)** TG content in the feces of mice. **(E)** TC content in the feces of mice. **(F)** Expression of NPC1L1 protein in mice jejunum. **(G)** Mice adipose tissue section (100 mice). *P < 0.05; **P < 0.01; ***P < 0.001; ****P < 0.0001.

Research has shown that HFD mice may have higher levels of inflammation than ND mice, including an increase in pro-inflammatory cytokines and related inflammatory proteins (Terzo et al., 2020; Kuerman et al., 2021; Chan et al., 2022). The occurrence of these inflammations may also be related to the fragile body state of mice, which made them more susceptible to colonization by pathogenic bacteria (Huang et al., 2023). To demonstrate the effects of L-27-2, L-14-1, and F203 on obesity-induced inflammation *in vivo*, after feeding HFD mice with lactic acid bacteria for 28 days, serum IL-6 levels in mice were detected by ELISA detection kit. Then, it was found that the serum IL-6 content of mice was significantly increased by HFD (*P* < 0.01), but the blood IL-6 of mice fed with L-27-2 and L-14-1 was significantly decreased (*P* < 0.01), and the IL-6 of mice fed with F203 was decreased, but not significant (*P* > 0.05) (Figure 7A). These results showed that L-27-2, L-14-1, and F203 could help alleviate increased weight gain by HFD and inflammation caused by obesity.

### 3.11 Lactic acid bacteria regulated cholesterol and triglycerides in the liver of mice fed the HFD

It has been found that the HFD can increase the content of total cholesterol TC and TG in the liver, and eventually lead to liver lipid deposition and even nonalcoholic liver disease (Recena Aydos et al., 2019; Ding et al., 2022). Therefore, in order to investigate the effects of L-27-2, L-14-1, and F203 on lipid accumulation in the liver of HFD mice induced by the HFD, the contents of TG and TC in the liver of mice were determined by ELISA. It was found that HFD could increase the content of TC and TG in the liver of mice, while feeding L-27-2 could decrease the content of TC (*P* < 0.05) (Figure 7B), and feeding L-27-2, F203 could decrease the content of TG in the liver of HFD mice (*P* < 0.05) (Figure 7C).

### 3.12 Lactic acid bacteria altered cholesterol and triglycerides in feces of mice fed the HFD

Studies have shown that accelerating the excretion of TC and TG from the body is helpful in reducing lipid deposition *in vivo*, which is manifested by the increase of TC and TG in feces (Kong et al., 2019). Therefore, in order to explore the ability of L-27-2, L-14-1, and F203 to metabolize lipids in obese mice induced by the HFD, the contents of TG and TC in the feces of mice were determined by ELISA. It was found that feeding L-27-2, L-14-1, and F203 could increase TC content in feces of mice fed with HFD (Figure 7E), but the TG metabolism in feces did not increase significantly (*P* > 0.05) (Figure 7D). Therefore, L-27-2, L-14-1, and F203 may be helpful for TC metabolism under the HFD.

### 3.13 Lactic acid bacteria increased NPC1L1 expression in the gut of mice fed the HFD

Lack of NPC1l1 has an effect on cholesterol metabolism, and inhibition and lack of NPC1L1 has been shown to protect mice from obesity induced by the HFD (Jia et al., 2021; Meng et al., 2022). Therefore, in order to investigate the effects of L-27-2, L-14-1, and F203 on the expression of NPC1L1 protein in the intestine, the expression of NPC1L1 in the jejunum of mice was measured by ELISA. The results showed that L-27-2, L-14-1, and F203 could reduce the expression of NPC1L1 protein induced by HFD (*P* > 0.05) (Figure 7F), and inhibition of this protein might be the specific pathway of L-27-2, L-14-1, and F203 acting on cholesterol metabolism.

### 3.14 Lactic acid bacteria reduced inflammatory levels of adipose tissue in mice fed the HFD

Studies have shown that in obese mice fed the HFD, fat accumulates significantly in the abdominal cavity and is accompanied by inflammation (van der Heijden et al., 2015; Airaksinen et al., 2018). Therefore, in order to explore the effects of L-27-2, L-14-1, and F203 on fatty tissue inflammation in obese mice induced by the HFD, white fat in the abdomen of mice was taken for pathological section, and it was found that fatty inflammation in HFD mice was alleviated to varying degrees after feeding L-27-2, L-14-1, and F203, of which L-27-2 had the best effect and the average diameter of adipocytes was smaller and fewer inflammatory cells were observed (Figure 7G). This showed that L-27-2, L-14-1, and F203 have certain inhibitory effects on fatty inflammation produced by the HFD.

### 3.15 Reversal of intestinal flora imbalance induced HFD by lactic acid bacteria

**FIGURE 8:**
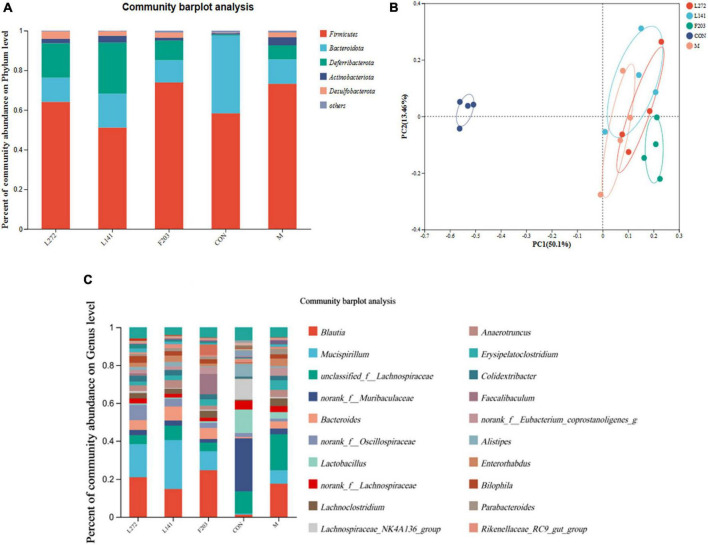
Microbial diversity of colon contents in mice. **(A)** Microbial diversity of mice colon contents (phylum level). **(B)** Microbial diversity of mice colon contents (phylum level). **(C)** Microbial diversity of mice colon contents (genus level).

In this study, it was found that the HFD could reduce the number of Bacteroides and increase the abundance of Firmicutes in the gut of mice, but feeding L-27-2, L-14-1, and F203 could slow this change, thus reducing the number of Firmicutes and increasing the number of Bacteroidetes in the gut of HFD mice (Figure 8A). Using PCoA diagram analysis, it was found that feeding L-27-2, L-14-1, and F203 could change the intestinal microbial diversity of mice, among which L-27-2 and F203 were significantly different from HFD mice, while L-14-1 was slightly different, and the intestinal microbial diversity of mice fed L-27-2, L-14-1, and F203 increased significantly (Figure 8B). At a subordinate level, the HFD might increase the number of *Erysipelatoclostridium* in the gut of mice. Feeding L-27-2, L-14-1, and F203 might reduce the accumulation of *Erysipelatoclostridium* and *Lachnoclostridium* in mice fed the HFD. However, *Erysipelatoclostridium* enrichment in the body was associated with the occurrence of many diseases (Milosavljevic et al., 2021), and *Lachnoclostridium* has been shown to be associated with rectal adenoma and cancer (Liang et al., 2020). In addition, *Faecalibaculum*, as a kind of short-chain fatty acid-producing bacteria, has been shown to have the effect of preventing tumor growth (Zagato et al., 2020). This study found that *Faecalibaculum* was clearly enriched in mice fed L-27-2 and F203, indicating the positive effect of lactic acid bacteria on the intestinal flora of mice (Figure 8C). These results indicated that L-27-2, L-14-1, and F203 have effects on intestinal microorganisms in mice, which was related to the increasing SCFA, lipid metabolism, and the decreasing of pathogenic bacteria colonization in mice. All results were uploaded to NCBI with SRA number as PRJNA1050058.

## 4 Discussion

The effects of lactic acid bacteria on cholesterol metabolism and pathogenic bacteria in cats were studied by isolating various lactic acid bacteria from different cat feces. By screening 85 cat feces samples, three probiotic strains with cholesterol degradation capacity and pathogenic bacteria inhibition capacity were finally screened. After 16S rRNA sequencing analysis, it was found that these three strains were *L. plantarum, Pediococcus* sp., and *E. faecium*, respectively. It has been reported that lactic acid bacteria have the ability to reduce fat, and most probiotics have the ability to inhibit the growth of intestinal pathogenic bacteria (Kuebutornye et al., 2020). The three strains screened in this study have the ability to lower cholesterol by more than 15% and antibacterial circle radius greater than 15 mm. In addition, good probiotics need to evaluate whether the strain can withstand the receptor’s internal environment, because the extreme environment in the gastrointestinal tract is closely related to its growth capacity, acid resistance and bile salt resistance. After entering the gastrointestinal tract, probiotics need to tolerate the acidic environment with low pH in the stomach first, and then the effect of intestinal bile salt on it (Philipp, 2011; Feyereisen et al., 2020). After that, it is colonization of the strain in the intestine. Probiotics without good colonization capacity cannot play a long-term role in the gut, and cannot compete with other strains, meaning they cannot improve the body’s function as much as they can (Hecht et al., 2016). The three strains screened in this study could well tolerate the acidic environment with pH 2 and the bile salt environment of 0.6%. Among them, survival rates of L-27-2, L-14-1, and F203 were 93.71%, 96.80%, and 70.17% at pH 2, and survival rates of L-27-2, L-14-1, and F203 were 93.71%. In addition, carbohydrate utilization capacity of lactic acid bacteria are also one of the main methods to judge the function of lactic acid bacteria (Singhvi et al., 2018). According to the regulations for the use of lactic acid bacteria, all lactic acid bacteria must be evaluated for safety before use (Selvin et al., 2020). The three strains isolated in this experiment were all safe strains as defined by the Chinese Ministry of Agriculture and Rural Affairs. And the three strains were sensitive to clinically used antibiotics and had no antibiotic resistance.

In this study, we compared the effects of HFD and lactic acid bacteria on HFD mice, including the effects on lipid-related markers and gut microbiology, we did this 28 days after the start of the study and found that feeding the HFD diet for 28 days induced an increase in body weight in the mice, which is consistent with Li et al.’s (2020) finding that the HFD diet induced obesity in mice. In addition, supplementation with lactic acid bacteria was found to slow the process of obesity, including body weight, fat mass and inflammation levels, in studies of obese animals (Kang et al., 2022), which is consistent with the findings of this study. In this experiment, it was found that supplementation of three lactic acid bacteria strains to mice fed an HFD could slow or inhibit the obesity process to varying degrees, reduce body weight and reduce abdominal fat accumulation, and it was also found that Lactobacillus increased lipid metabolism in the mice, including the reduction of TC and TG in the blood, and also alleviated inflammation induced by the HFD in mice, with the varying effects toward alleviating effects of HFD seen in L-27-2, L-14-1, and F203. Lactic acid bacteria were also found to reduce adipose tissue inflammation and lipid droplet accumulation in adipocytes. This resulted in a lipid-lowering effect, consistent with the phenomenon observed in adipocytes by Suárez-Zamorano et al. (2015).

Previous studies have found that lactic acid bacteria are mostly known for their lipid-lowering effects by affecting the gut microbes and thus lipid metabolism in mice (Kang et al., 2023). Therefore, the present experiment was conducted to investigate the possible effects of lactic acid bacteria by examining the changes in gut microorganisms, and it was found that lactic acid bacteria can cause significant changes in gut microorganisms, and these findings are consistent with the findings of Wang et al. (2020). Studies have shown that Firmicutes and Bacteroides are the dominant bacteria in the gut (Liu et al., 2021), and the increase in Firmicutes and decrease in Bacteroides may be a manifestation of obesity (Ley et al., 2005; Magne et al., 2020), but feeding L-27-2, L-14-1, and F203 could slow this change, thus reducing the number of Firmicutes and increasing the number of *Bacteroidetes* in the gut of HFD mice. In addition, this experiment also found that feeding lactic acid bacteria could cause a decrease in *Erysipelatoclostridium*, which to some extent attenuated the increase in obesity in HFD mice (Chen et al., 2023), and lactic acid bacteria also altered β-diversity in HFD mice, which is consistent with the results found by Yin et al. (2018). Taken together, these results suggest that the lactic acid bacteria strains isolated in the present experiment may achieve fat loss by altering the gut microbiota, thereby affecting intestinal cholesterol absorption, liver lipid metabolism, and the intestinal flora of mice, which was consistent with the former findings (Wang et al., 2021). More bacteria associated with SCFA production and lactic acid bacteria for the prevention of intestinal diseases were also found in the intestines of mice fed lactic acid bacteria, which were better able to resist the negative effects of a HFD and external pathogens (Den Besten et al., 2015). The anti-inflammatory effects of lactic acid bacteria found in this study may also be related to changes in the microbiological composition of the mouse gut, and although it has been claimed that lactic acid bacteria can affect inflammation levels (Levit et al., 2021). The exact anti-inflammatory mechanisms of lactic acid bacteria need to be deeply investigated in future.

## 5 Conclusion

**FIGURE 9:**
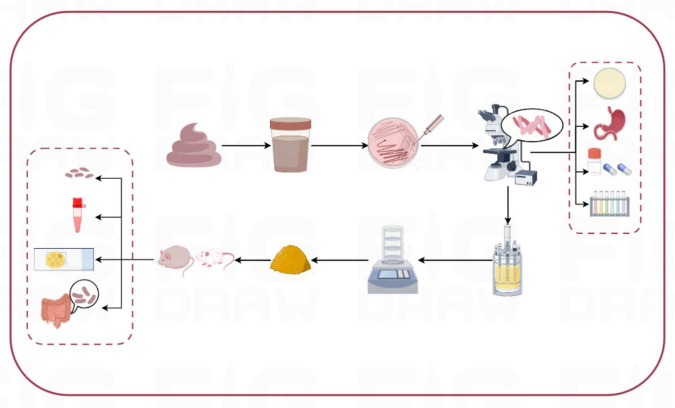
Experimental flow chart.

Three strains of lactic acid bacteria, separately named L-27-2, L-14-1, and F203, were isolated from healthy cat feces, and all showed good characterization *in vitro* in the test. The results showed that the three isolates had lipid-lowering ability *in vivo* and *in vitro*, and could be probiotics for improving obesity problem for pets in Figure 9.

## Data availability statement

The datasets presented in this study can be found in online repositories. The names of the repository/repositories and accession number(s) can be found below: https://www.ncbi.nlm.nih.gov/genbank/, OR919791; https://www.ncbi.nlm.nih.gov/genbank/, OR919790; https://www.ncbi.nlm.nih.gov/genbank/, OR919789; https://www.ncbi.nlm.nih.gov/, PRJNA1050058.

## Ethics statement

The animal studies were approved by the Laboratory Animal Ethical Committee and its Inspection of the Institute of Feed Research of CAAS. The studies were conducted in accordance with the local legislation and institutional requirements. Written informed consent was obtained from the owners for the participation of their animals in this study.

## Author contributions

SL: Conceptualization, Data curation, Formal analysis, Investigation, Methodology, Software, Writing – original draft, Writing – review & editing. YK: Data curation, Methodology, Writing – original draft. YZ: Formal analysis, Methodology, Writing – original draft. JS: Methodology, Writing – original draft. XW: Data curation, Project administration, Writing – review & editing. HT: Software, Validation, Writing – review & editing. ZW: Resources, Validation, Writing – review & editing. JW: Funding acquisition, Resources, Writing – review & editing. YGZ: Funding acquisition, Investigation, Writing – review & editing. BH: Funding acquisition, Resources, Writing – review & editing.
