# Supplementary material for: Characterization and potential lipid-lowering effects of lactic acid bacteria isolated from cats
Source: Front Microbiol. 2024 Apr 24;15:1392864. doi: 10.3389/fmicb.2024.1392864 (PMC11076690; doi:10.3389/fmicb.2024.1392864)
Supplement: Supplementary file 1 [file Table_1.DOCX]

Supplementary Material

# Supplementary Tables

| Strain number | Ring (mm)^a^ | Strain number | Ring (mm) | Strain number | Ring (mm) | Strain number | Ring (mm) |
| --- | --- | --- | --- | --- | --- | --- | --- |
| L-22-3 | ++ | L-47-2 | + | L-26-3 | + | L-11-4 | + |
| L-27-2 | ++ | L-47-1 | + | L-26-1 | + | L-11-2 | + |
| L2117 | ++ | L-46-2 | + | L-25-2 | + | L-1-1 | + |
| L-8-1 | ++ | L-46-1 | + | L-25-1 | + | L-10-3 | + |
| L-26-2 | ++ | L-45-3 | + | L-24-4 | + | L-10-2 | + |
| L-23-5 | ++ | L-45-1 | + | L-24-3 | + | L-10-1 | + |
| L-23-2 | ++ | L-44-3 | + | L-24-2 | + | F210 | + |
| L2121 | ++ | L-44-2 | + | L-24-1 | + | F209 | + |
| L2114 | ++ | L-44-1 | + | L-2-4 | + | F208 | + |
| L2106 | ++ | L-43-3 | + | L-23-4 | + | F207 | + |
| L-14-1 | ++ | L-43-2 | + | L-23-3 | + | F206 | + |
| L-13-1 | ++ | L-42-4 | + | L-23-1 | + | F205 | + |
| L-12-4 | ++ | L-42-3 | + | L-2-3 | + | F204 | + |
| L-11-1 | ++ | L-42-2 | + | L-22-2 | + | F202 | + |
| F203 | ++ | L-42-1 | + | L-22-1 | + | F201 | + |
| L-9-1 | ++ | L-4-2 | + | L-2-2 | + | F200 | + |
| L-8-3 | + | L-41-3 | + | L-21-3 | + | L-9-3 | + |
| L-8-2 | + | L-41-2 | + | L2120 | + | L-9-2 | + |
| L-7-2 | + | L-40-3 | + | L-21-2 | + | L-54-5 | + |
| L-7-1 | + | L-40-2 | + | L2119 | + | L-54-1 | + |
| L-6-3 | + | L-40-1 | + | L2118 | + | L-52-3 | + |
| L-6-2 | + | L-39-3 | + | L2116 | + | L-51-4 | + |
| L-6-1 | + | L-39-2 | + | L2113 | + | L-49-5 | + |
| L-54-6 | + | L-39-1 | + | L2112 | + | L-49-1 | + |
| L-54-4 | + | L-38-2 | + | L2111 | + | L-48-3 | + |
| L-54-3 | + | L-38-1 | + | L2110 | + | L-46-3 | + |
| L-54-2 | + | L-37-1 | + | L2109 | + | L-45-2 | + |
| L-53-4 | + | L-36-1 | + | L2108 | + | L-43-1 | + |
| L-53-3 | + | L-35-2 | + | L2105 | + | L-4-3 | + |
| L-53-2 | + | L-35-1 | + | L2104 | + | L-41-1 | + |
| L-53-1 | + | L-34-5 | + | L2103 | + | L-4-1 | + |
| L-5-3 | + | L-34-4 | + | L2102 | + | L-36-2 | + |
| L-52-5 | + | L-34-2 | + | L2101 | + | L-34-3 | + |
| L-52-4 | + | L-34-1 | + | L-2-1 | + | L-30-1 | + |
| L-52-2 | + | L-3-4 | + | L-20-3 | + | L-25-3 | + |
| L-52-1 | + | L-33-3 | + | L-20-1 | + | L2115 | + |
| L-5-2 | + | L-33-2 | + | L-19-3 | + | L-21-1 | + |
| L-51-6 | + | L-33-1 | + | L-19-2 | + | L2107 | + |
| L-51-5 | + | L-3-3 | + | L-18-3 | + | L-20-2 | + |
| L-51-3 | + | L-32-3 | + | L-18-2 | + | L-19-1 | + |
| L-51-2 | + | L-32-2 | + | L-18-1 | + | L-12-2 | + |
| L-51-1 | + | L-32-1 | + | L-17-2 | + | L-11-3 | + |
| L-5-1 | + | L-3-2 | + | L-17-1 | + | L-12-3 | + |
| L-50-5 | + | L-31-3 | + | L-16-2 | + | L-12-1 | + |
| L-50-4 | + | L-31-2 | + | L-16-1 | + | L-1-2 | + |
| L-50-3 | + | L-31-1 | + | L-15-1 | + | L-28-2 | + |
| L-50-2 | + | L-3-1 | + | L-14-2 | + | L-28-1 | + |
| L-50-1 | + | L-30-4 | + | L-13-5 | + | L-27-1 | + |
| L-49-4 | + | L-30-3 | + | L-13-4 | + | L-48-2 | + |
| L-49-3 | + | L-30-2 | + | L-13-3 | + | L-48-1 | + |
| L-49-2 | + | L-29-3 | + | L-13-2 | + | L-47-3 | + |
| L-48-5 | + | L-29-2 | + | L-1-3 | + |  |  |
| L-48-4 | + | L-29-1 | + | L-12-5 | + |  |  |

**Supplementary Table 1.** Calcium dissolving circle of 211 lactic acid bacteria strains (mm). ^a^ +:≤ 1 mm; ++: >1 mm

| Strain number | Colony characteristic cell shape | Cell shape  (×1000） | Gram staining |
| --- | --- | --- | --- |
| F203 | Milky white, round and convex, smooth surface | spherical | G+ |
| L-8-2 | Milky white, radial, uneven surface | rod-shaped | G+ |
| L-11-1 | Milky white, round and convex, smooth surface | thin rod-shaped | G+ |
| L-12-4 | Milky white, round and convex, smooth surface | rod-shaped | G+ |
| L-13-1 | Milky white, round and convex, smooth surface, large colonies | spherical | G+ |
| L-14-1 | Milky white, round and convex, smooth surface, small colonies | rod-shaped | G+ |
| L-9-1 | Milky white, round and convex, smooth surface | rod-shaped | G+ |
| L-22-3 | Milky white, round and convex, smooth surface | spherical | G+ |
| L-23-2 | Milky white, round and convex, smooth surface | spherical | G+ |
| L-23-5 | Milky white, round and convex, smooth surface | rod-shaped | G+ |
| L-26-2 | Milky white, round and convex, smooth surface | spherical | G+ |
| L-27-2 | Milky white, round and convex, smooth surface | rod-shaped | G+ |
| L2106 | Milky white, round and convex, smooth surface | thick rod-shaped | G+ |
| L2114 | Milky white, round and convex, smooth surface | rod-shaped | G+ |
| L2117 | Milky white, round and convex, smooth surface | rod-shaped | G+ |
| L2121 | Milky white, round and convex, smooth surface | rod-shaped | G+ |

**Supplementary Table 2.** Gram staining and microscopic morphology of 16 lactic acid bacteria strains
